# Variceal bleeding due to idiopathic portal vein thrombosis in a 15‐year‐old boy

**DOI:** 10.1002/ccr3.2313

**Published:** 2019-07-20

**Authors:** Dario Dilber, Dubravko Habek, Zlatko Hrgović, Jasna Čerkez Habek, Marina Gradišer

**Affiliations:** ^1^ Internal medicine Department County Hospital Čakovec Čakovec Croatia; ^2^ Gynecology Department University Hospital “Sv.Duh” Zagreb Croatia; ^3^ Uniklinik fur Pediatrie Frankfurt/Main Deutschland; ^4^ Cardiology Department University Hospital “Sv.Duh” Zagreb Croatia

**Keywords:** child, idiopathic, portal hypertension, portal vein thrombosis

## Abstract

Portal vein thrombosis is an important cause of portal hypertension in the pediatric population. It is a rare and potentially fatal condition with diverse underlying pathology. A successfully managed case, without an identified etiology, is reported herein.

## 1 INTRODUCTION

The risk of venous thromboembolism (VTE) is substantially lower in children compared with adults.^1^ Portal vein thrombosis (PVT) as a manifestation of VTE may occur in children with underlying risk factors such as malignancy, inherited thrombophilia, infections, splenectomy, sickle cell disease, chemotherapy, or the presence of antiphospholipid antibodies. Furthermore, it can present as a complication following pediatric liver transplantation.^2^, ^3^, ^4^, ^5^, ^6^, ^7^, ^8^, ^9^

We report a case of an idiopathic portal vein thrombosis in a child, presenting with portal hypertension, esophageal and rectal varices, splenomegaly, and gastrointestinal bleeding with unconfirmed etiology in known medical conditions.

## 2 CASE REPORT

A 15‐year‐old Caucasian boy presented to the Emergency Department (ED) of the Uniklinik für Pädiatrie, Frankfurt/Main with history of intensifying upper abdominal pain in the last 3 weeks and acute onset of bright red hematemesis. He had no serious medical conditions in previous medical history except lactose intolerance for the last 2 years and tonsilitis in two subsequent years and was taking no medications. Furthermore, the patient had no medical history of splenomegaly, anemia, or thrombocytopenia prior to admission and had no evidence of direct portal vein trauma in early childhood such as omphalitis or umbilical vein catheterization. There was no history of traveling to endemic areas with schistosomiasis, such as Egypt or tropical areas. Family history was unremarkable with no acute thrombotic events, and two healthy older sisters.

### 2.1 Clinical findings

At presentation, he was hemodynamically stable, pale, and without pain. Physical examination revealed splenomegaly, and no other pathological findings other than tachycardic heart rhythm on heart auscultation, which was attributed to patient anxiety.

### 2.2 Diagnostic assessment

The patients' blood pressure (BP) was 115/80 mm Hg, pulse was 98 bpm, pulse oximeter showed 98% oxygenation, and his body temperature was 36.7°C. His body mass index was 21.6 kg/m^2^(75 percentile). Routine laboratory workup showed no abnormal findings other than anemia (hemoglobin level of 8.8 mg/dL), and platelet levels were 90 000/mm^3^. Cardiac pathology was ruled out with echocardiography, chronic inflammatory conditions were ruled out by C‐reactive protein (CRP) and sedimentation rate (SE) levels which were in normal ranges, and tumor markers were obtained to exclude malignancy (alpha‐fetoprotein, CA 19‐9, carcinoembryonic antigen, human chorionic gonadotropin). There were no laboratory findings consistent with pancreatitis as a cause of upper abdominal pain; lipase levels were within normal ranges in consecutive tests.

Ultrasound of the abdomen revealed absent flow in the portal vein by color Doppler imaging due to thrombotic material at the confluence of the right and left bundle branch with dilatation of proximal portal vein of 14 mm in diameter and hepatic venous pressure gradient (HVPG) greater than 10 mm Hg, splenomegaly of 18 × 12 cm with dilated splenic vein size to 9 mm and normal flow, and no visible pathology of the liver parenchyma or bladder, no organ or extrahepatic masses, or presence of portal vein cavernoma, with only slightly enlarged liver of 0.5 cm over the upper limit. Magnetic resonance angiography (MRA) confirmed PVT. Liver function tests were normal including bilirubin levels, GGT, and AST/ALT, and chronic parenchymal liver disease was excluded (negative serology of hepatotropic viruses—HBV, HCV, CMV, and EBV; FibroScan Score F0; protein; albumin; fibrinogen levels; and lipid profile were in normal ranges).

Ceruloplasmin and blood and urine copper levels as well as blood iron and ferritin levels were within normal ranges. Coagulation tests were within normal ranges, and thrombophilia was excluded, test including Antithrombin III, Protein S, Protein C, Leiden mutation, prothrombin gene mutation, MTHFR, and PAI‐1 was made.

### 2.3 Therapeutic interventions

**Figure 1:**
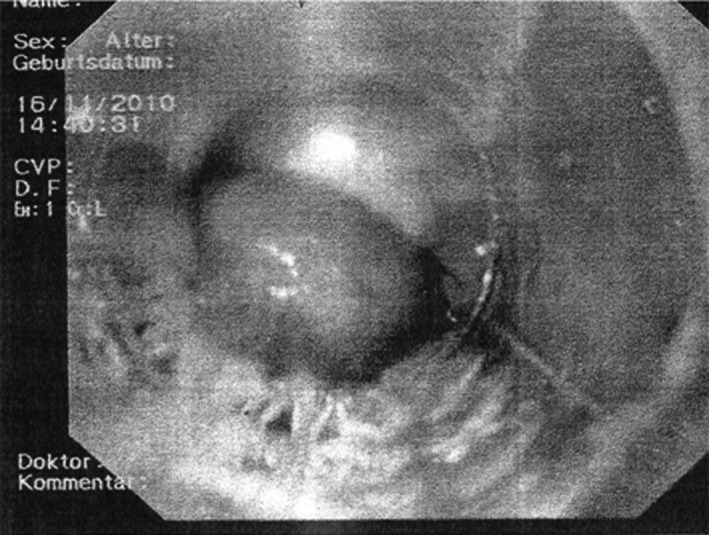
Ligation of esophageal varices

Urgent esophagoscopy revealed varices in the middle third of the esophagus as the source of bleeding and was described as grade 2 as tortuous veins occupied less than 1/3 of esophageallumen. Ligation of the varices with a rubber band ligation device and endoscopic injection sclerotherapy with 5% ethanolamine oleate was performed (Figure 1). Patient was started on peroral propranolol, maximal PPI doses of omeprazol (40 mg BD iv), and octreotide pump, set at 2.5 mcg/hr for a total of 3 days. Low molecular weight heparin or peroral warfarin in the indexed hospitalization was not administered after cessation of variceal bleeding. Patient was discharged with omeprazole at peroral dose of 40 mg twice a day, propranolol at peroral dose of 10 mg three times a day, and peroral iron substitution with dextriferron at peroral dose of 100 mg once a day.

### 2.4 Follow‐up and outcomes

At the follow‐up outpatient visit after 4 weeks, the blood count was within normal ranges and the patient was symptom‐free. Follow‐up videoendoscopy revealed ligated varices of the esophagus with no signs of recent bleeding, and mucosal erythema and edema consistent with acute gastritis confirmed with histological findings of biopsy specimens taken at endoscopy. Ileocolonoscopy revealed rectal varices with no other pathological findings. Control ultrasound of the abdomen revealed absent flow in the portal vein by color Doppler imaging with dilatation of the proximal portal vein of 12 mm in diameter. As no current variceal bleeding was present, the patient was started on peroral anticoagulant therapy with warfarin, with a target INR of 2‐2.5 until the next ambulatory visit scheduled in 3 months.

Consent of the child and parental permission was obtained regarding the reporting of the case.

## 3 DISCUSSION

Portal vein obstruction is a relatively rare condition with an overall incidence of 0.05%‐0.5% in autopsy studies.^2^ Portal vein thrombosis is an important cause of portal hypertension (PH) in the pediatric population with different etiology, management, and outcome, as compared to the adult population. Portal hypertension is hypertension (high blood pressure) in the hepatic portal system—made up of the portal vein and its branches, and is defined as a hepatic venous pressure gradient. PH can be extrahepatic (prehepatic obstruction), intrahepatic or postsinusoidal, and cirrhotic and noncirrhotic. Risk factors associated with PVT include exchange transfusions, hypercoagulability states, cirrhosis, congenital portal vein malformations, umbilical vein catheterization, omphalitis and sepsis, pancreatitis, trauma or manipulation of the portal vein, malignancy and abdominal operations, or a surgical diagnosis such as acute appendicitis.^2^, ^3^, ^4^, ^5^, ^6^, ^7^, ^8^, ^9^ Children are less frequently exposed to acquired prothrombotic risk factors (eg, hormone replacement therapy, malignancy, surgery) and are at lower risk for thromboembolic events as compared to adults due to the fact that plasma concentrations of all vitamin K‐dependent factors, almost all contact factors, and the capacity to generate thrombin are decreased throughout childhood, while the capacity to inhibit thrombin is enhanced due to increased plasma concentrations of the thrombin inhibitor alpha‐2‐macroglobulin.^10^

In this case, a positive causative etiology was not identified; however, the abovementioned differential diagnoses were not confirmed. The cause of abdominal pain cannot be explained directly by portal vein thrombosis but it can be a result of abdominal organomegaly and can be attributed to abdominal visceral pain, induced by stretching of hollow viscera or parenchymal viscera walls, and is a symptom of acute PVT as a prehepatic cause of portal hypertension. This report describes a symptomatic portal vein thrombosis with unconfirmed causative etiology in known medical conditions and is important as it emphasizes the paramount importance of a detailed investigation of possible underlying disorders in such conditions. There are a few previously reported cases in the literature with a similar clinical presentation of portal vein thrombosis in a child, but with identified risk factors for thrombosis, such as thrombophilia.^11^

This report has limitations. We did not exclude intrinsic liver disease with biopsy of the liver due to unwillingness of the patient and his family to proceed with further invasive diagnostic methods. Although we cannot definitely exclude intrahepatic causes of portal hypertension, based on noninvasive diagnostic methods including radiological findings and laboratory workup findings, we can make a presumption of idiopathic portal vein thrombosis in our reported case.

Further studies are warranted due to complexity of pathogenic mechanisms of thrombophilic disorders which is a typical multifactorial disease, involving both genetic and circumstantial risk factors that affect the delicate balance between procoagulant and anticoagulant states.

## CONFLICT OF INTEREST

None.

## AUTHORS' CONTRIBUTIONS

DD: is a corresponding author and involved in design and drafting.

DH: involved in data requisition, revision, and final approval.

ZH: involved in data interpretation and revision.

JČH: involved in drafting and revision.

MG: involved in revision and final approval.
